# Evolution, phylogenetic distribution and functional ecology of division of labour in trematodes

**DOI:** 10.1186/s13071-018-3241-6

**Published:** 2019-01-04

**Authors:** Robert Poulin, Tsukushi Kamiya, Clément Lagrue

**Affiliations:** 1 Department of Zoology, University of Otago, Dunedin, New Zealand; 2 Department of Ecology and Evolutionary Biology, University of Toronto, Toronto, Canada; 3 Department of Biological Sciences, University of Alberta, Edmonton, Canada

**Keywords:** Caste, Cercariae, Fitness, Multiple infections, *Philophthalmus*, Rediae, Sociality, Soldiers, Trade-off

## Abstract

Division of labour has evolved in many social animals where colonies consist of clones or close kin. It involves the performance of different tasks by morphologically distinct castes, leading to increased colony fitness. Recently, a form of division of labour has been discovered in trematodes: clonal rediae inside the snail intermediate host belong either to a large-bodied reproductive caste, or to a much smaller and morphologically distinct ‘soldier’ caste which defends the colony against co-infecting trematodes. We review recent research on this phenomenon, focusing on its phylogenetic distribution, its possible evolutionary origins, and how division of labour functions to allow trematode colonies within their snail host to adjust to threats and changing conditions. To date, division of labour has been documented in 15 species from three families: Himasthlidae, Philophthalmidae and Heterophyidae. Although this list of species is certainly incomplete, the evidence suggests that division of labour has arisen independently more than once in the evolutionary history of trematodes. We propose a simple scenario for the gradual evolution of division of labour in trematodes facing a high risk of competition in a long-lived snail host. Starting with initial conditions prior to the origin of castes (size variation among rediae within a colony, size-dependent production of cercariae by rediae, and a trade-off between cercarial production and other functions, such as defence), maximising colony fitness (*R*_0_) can lead to caste formation or the age-structured division of labour observed in some trematodes. Finally, we summarise recent research showing that caste ratios, i.e. relative numbers of reproductive and soldier rediae per colony, become more soldier-biased in colonies exposed to competition from another trematode species sharing the same snail, and also respond to other stressors threatening the host’s survival or the colony itself. In addition, there is evidence of asymmetrical phenotypic plasticity among individual caste members: reproductives can assume defensive functions against competitors in the absence of soldiers, whereas soldiers are incapable of growing into reproductives if the latter’s numbers are reduced. We conclude by highlighting future research directions, and the advantages of trematodes as model systems to study social evolution.

## Background

Division of labour, i.e. the performance of different tasks by different units, underpins the evolution and organisation of complex modular systems, from cell and tissue differentiation in multicellular organisms to worker specialisation on assembly lines in modern factories [1, 2]. It is also a key feature in many social animals, where colonies consist of a reproductive caste and various other morphologically distinct castes that perform different functions for the colony’s benefit [3, 4]. Increased efficiency in task performance through functional specialisation and the resulting improvement in colony success are the advantages of division of labour that drive the evolution of social animals. We generally think of colonies of eusocial insects, like honey bees, ants and termites as examples of division of labour in the animal kingdom; however, division of labour among distinct castes has evolved in multiple other taxa where individuals form groups of clones or close kin [5].

**Fig. 1:**
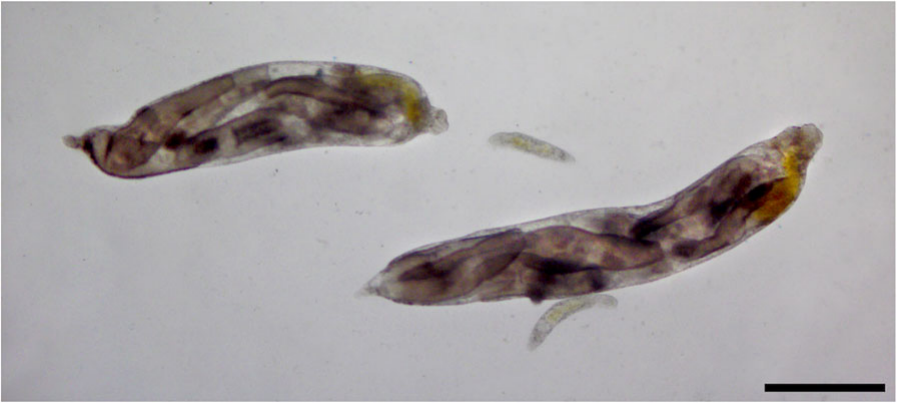
Rediae of *Philophthalmus* sp. extracted from their snail intermediate host, *Zeacumantus subcarinatus*. Shown are two large reproductive rediae, each filled with several cercariae, and two small soldiers. Note that soldiers are smaller than cercariae. *Scale-bar*: 500 μm

Recently, division of labour has been documented in parasitic trematodes [6, 7]. Trematodes have complex life-cycles, beginning typically with a larva hatching from an egg and infecting a snail first intermediate host [8]. Within the snail, the parasite multiplies asexually, resulting in a colony of clonal parthenitae, which depending on the species will be either sporocysts or rediae. These are sac-like individuals containing stem cells, the main difference between them being that rediae possess a mouth, pharynx and gut whereas sporocysts do not. Whatever the type of parthenitae involved, they produce and release dispersal stages known as cercariae, which leave the snail to seek the parasite’s next host and continue the life-cycle. It is now clear that in several trematode species that have rediae, the latter come in two distinct morphs (Fig. 1). Large rediae, hereafter referred to as the reproductive caste, produce cercariae; they have relatively small mouthparts and are mostly immobile. In contrast, another morphologically distinct caste of smaller-bodied and much more mobile ‘soldiers’ have relatively large mouthparts and are specialised for colony defence, i.e. aggressive interactions against other trematode competitors [6, 7, 9]. Soldiers attach to competitors with their mouthparts and kill them, within minutes or hours [7]. They are disproportionately abundant in parts of the snail body where host entry by other trematodes can occur. Although this could simply be the outcome of passive movement by small rediae and their accumulation near the head and foot of the host snail [10], the presence of soldiers in these locations could act as a barrier against invasion.

In some species, it appears clear that the small soldiers never develop into larger reproductives (see Phenotypic plasticity of caste members below), and that this is a true case of division of labour between two distinct castes. In others, the division of labour may instead be age-structured, with younger and smaller rediae acting as soldiers before they grow into reproductive individuals [11, 12]. In fact, the mere existence of castes and division of labour in trematodes is not without controversy; Galaktionov et al. [12] provide an alternative interpretation based on allometric growth from young to older rediae and other age-related differences in mobility and function.

The discovery of trematodes with division of labour sparked much interest [13]. Here, we summarise the research conducted on these trematodes since their original discovery, focusing on three key aspects. First, we examine the distribution of species with documented division of labour across the trematode phylogeny. Secondly, we highlight the common external factors linking these species as possible clues to the conditions favouring the evolution of division of labour, and also propose a simple scenario for the stepwise evolution of distinct castes with specialised functions. Finally, we review recent research findings on the dynamics of trematode colonies with division of labour, both in terms of their ability to alter their ratio of soldiers to reproductives, and also with respect to within-caste phenotypic plasticity of individuals. This synthesis concludes with a short list of open questions for future research, and also emphasises the untapped value of trematodes as model systems for evolutionary biology.

## Division of labour across the trematode phylogeny

Since the original reports of division of labour in trematodes [6, 7], there have been several other studies documenting the presence of distinct castes in other trematode species [11, 14–16]. The evidence for division of labour always involves a detailed morphological comparison highlighting the distinct physical traits of the two castes, often accompanied by behavioural observations confirming that the smaller soldiers conduct attacks on non-colony members at a much higher frequency than reproductives, and (ideally) some evidence that small soldiers do not grow into large reproductive rediae. To date, division of labour has been confirmed in 15 trematode species, belonging to at least 9 genera and to 3 families: Himasthlidae (formerly a subfamily of Echinostomatidae), Philophthalmidae and Heterophyidae (Table 1).

**Table 1:** Trematode species in which division of labour (reproduction *versus* defence) has been demonstrated and/or inferred

Family	Species	Snail host	Location	Evidence	Reference
Himasthlidae	*Himasthla* sp. B	*Cerithidea californica*	California	M, B	[6]
	*Himasthla rhigedana*	*C. californica*	California	M, B	[14]
	*Himasthla elongata*	*Littorina littorea*	Denmark	M, B	[10]
	*Acanthoparyphium spinulosum*	*C. californica*	California	M, B	[14]
	*Acanthoparyphium* sp. I	*Batillaria attramentaria*	Japan	M, B	[13]
	*Acanthoparyphium* sp.	*Zeacumantus subcarinatus*	New Zealand	M	C. Lagrue (unpubl. data)
Philophthalmidae	*Philophthalmus* sp.	*Zeacumantus subcarinatus*	New Zealand	M, B	[7]
	*Cloacitrema michiganensis*	*C. californica*	California	M, B	[14]
	*Parorchis acanthus*	*C. californica*	California	M, B	[14]
	Philophthalmid sp. I	*B. attramentaria*	Japan	M, B	[13]
	Philophthalmid sp. II	*B. attramentaria*	Japan	M, B	[13]
Heterophyidae	*Euhaplorchis californiensis*	*C. californica*	California	M, B	[15]
	*Phocitremoides ovale*	*C. californica*	California	M	[15]
	*Pygidiopsoides spindalis*	*C. californica*	California	M	[15]
	*Strictodora hancocki*	*C. californica*	California	M, B	[15]

*Abbreviations*: *M* morphological characterisation of two distinct castes, *B* behavioural demonstration of greater defensive role of small soldiers

In addition to the species listed in Table 1, there are probably several others in which two redial morphs of greatly different sizes have been observed and reported, e.g. [17, 18], but not explicitly interpreted as members of different castes. For example, O’Dwyer et al. [19] described larval stages of trematodes from the intertidal snails *Austrolittorina antipodum* and *A. cincta* in New Zealand. These included *Parorchis* sp. (Philophthalmidae), in which the authors report the presence of two types of rediae, a small one with a relatively large pharynx and locomotor appendages, and a large one containing cercariae and possessing a small pharynx and no distinct locomotor appendage. As many of the small rediae did not have germinal masses, this may well have been another example of division of labour with separate reproductive and soldier castes. Furthermore, there must be many other cases where division of labour went totally unnoticed, with the smaller soldiers either overlooked or mistakenly assumed to represent earlier developmental stages of ‘standard’ rediae and therefore not even mentioned.

In contrast, studies of two species in the genus *Echinostoma* (Echinostomatidae) that were specifically looking for evidence of division of labour have failed to find any [15, 20]. Therefore, there is variability within the superfamily Echinostomatoidea (which includes the families Echinostomatidae and Himasthlidae), with some species having distinct functional and morphological castes, and others showing no evidence of division of labour whatsoever.

There may also be intraspecific variation in the manifestation of division of labour among populations of the same trematode species. For instance, if conditions in a particular locality do not favour division of labour and make it costly compared to a colony of morphologically homogeneous rediae, the frequency of caste formation genes in the local trematode population would be expected to decline. Thus, evidence for the existence or absence of division of labour in a given species should ideally come from multiple sampled populations.

With this caveat in mind, it is still informative to speculate on the phylogenetic origins of division of labour based on its currently known taxonomic distribution. Molecular phylogenetic relationships among trematode families indicate that Himasthlidae and Philophthalmidae are closely related taxa, whereas the Heterophyidae family occupies a different branch of the trematode tree [21, 22]. Nevertheless, Himasthlidae and Philophthalmidae are not true sister taxa [22], and therefore at this stage we tentatively suggest that division of labour has had three independent evolutionary origins in trematodes, one in each of the above three families.

## Adaptive evolution of division of labour in trematodes

The benefit of division of labour is an increased efficiency in task performance through functional specialisation, which in turn leads to greater colony success, i.e. higher output of cercariae over the lifetime of the colony, under certain conditions. Some basic conditions have been proposed as highly favourable to the evolution of division of labour in trematodes [6]. First, natural selection may favour division of labour in trematode species with rediae that face a high risk of invasion. In other words, a specialised defensive caste may be favoured in trematodes whose snail intermediate host is used by a species-rich guild of trematodes with relatively high prevalences of multiple infections. This situation would lead to any given colony facing a high probability of competition from another trematode colony in the same snail. In interspecific competition, species with rediae but no division of labour are generally dominant over species with sporocysts [23]; having a specialised soldier caste can only enhance their competitive dominance. Second, division of labour should be favoured in trematodes exploiting a long-lived snail host. There may be little to gain by investing in the defence of an ephemeral resource, whereas a long-lived host may be worth defending against other parasites trying to establish their own colonies. The species in Table 1 seem to meet these conditions; for instance, the snails *Cerithidea californica* and *Zeacumantus subcarinatus* live > 8 years and are exploited by multiple trematode species, some of which reach high prevalence. The species in Table 1 are also all marine trematodes. However, there is no *a priori* reason why division of labour should be favoured in marine *versus* freshwater habitats. The fact that all currently known trematodes with division of labour are marine species may be due to study bias, or perhaps marine snails tend to live longer than freshwater ones, which would promote the evolution of division of labour.

Like modern-day trematode species without division of labour [8], clonal trematode colonies prior to the origin of distinct castes within snail hosts likely consisted of roughly identical rediae, all capable of producing cercariae and of some aggression toward competitors. The following basic principles must have applied in these colonies: (i) there existed some variability in size among rediae within a colony; (ii) larger rediae could produce more cercariae than smaller ones; and (iii) there was a trade-off between cercarial production and any other function that a redia could fulfil, such as defence against competitors. These are either already known or highly plausible aspects of the biology of trematode rediae, and, in addition to reasonably frequent sharing of the snail host with a competitor, they were the original conditions under which a specialised defensive became favoured by selection.

So how does division of labour affect parasite fitness and favour the evolution of this social structure? The redial stage of trematode parasites in snails is functionally similar to microparasites because they are clones that multiply within their host. Fitness of microparasites of a given clone, commonly known as the basic reproductive number (*R*_0_), is simply expressed as the product of parasite transmission per unit of time (*T*) and duration of infection (*D*):

$$ {R}_0=T\times D $$

Any parasite trait that increases this quantity should, in theory, be favoured by natural selection. Because host resources are finite and required for cercarial production (i.e. for trematode transmission to their next hosts), division of labour is likely a double-edged sword for transmission. Its cost lies in the fact that the production of a non-reproductive caste takes resources away from reproduction. In the presence of a competitor, this cost may be offset by removal/suppression of the competitor which would otherwise take resources away from the focal parasite. Note that the benefit of division of labour on transmission rate *per se* does not have to be greater than the cost, but its benefit on fitness as a whole (*R*_0_) does. Furthermore, division of labour could positively impact the duration of infection, another component of trematode colony fitness in the intermediate snail host. For instance, if soldiers feed mostly on competitors and less on host tissue, they may not contribute as much to the deterioration of host health; they would also make the colony’s competitive exclusion from the host by the rival parasite less likely. Both these factors can prolong the colony’s duration of host exploitation. Here, we just present the basic *R*_0_ condition (i.e. parasite fitness in a fully susceptible host population) to shed light on parasite fitness components influenced by division of labour. To fully understand its evolution, we would need to model the population dynamics of trematodes, for example using the adaptive dynamics approach, which would require us to make assumptions about numerous colony and population level parameters. This is beyond the scope of this review.

**Fig. 2:**
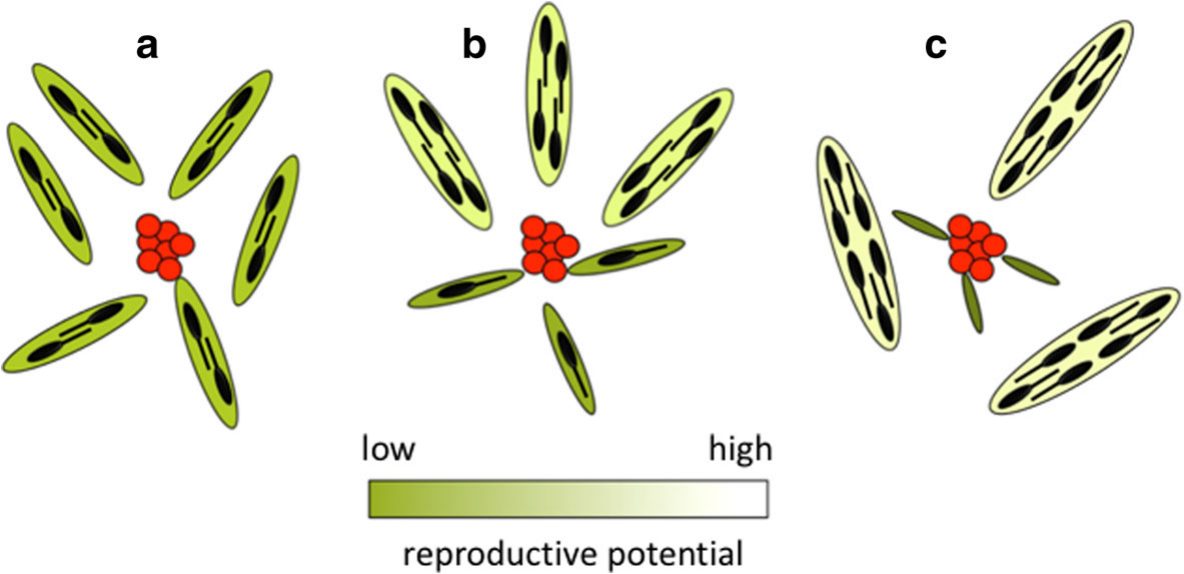
Three stages in the evolution of division of labour in trematode colonies. The total number of rediae (flattened ellipses) per colony and their total biomass, represented by their combined surface area, remain unchanged. Similarly, the size of the competing colony of sporocysts of a different species (red clump) also remains constant. A redia’s potential production of cercariae (black shapes) is indicated by the shading, with darker greenish shades representing low reproductive potential but high defensive ability, and *vice versa*. **a** No functional or morphological differentiation among rediae. **b** Some rediae compromise their cercarial production to invest more in attacks against the competitor, whereas others have limited their defensive duties to focus on growth and cercarial output. **c** Full separation of functions between specialised soldiers and specialised reproductives. Note that colony success increases by 50%, from a total of 12 cercariae per unit time (**a**) to 18 cercariae (**c**)

In practice, trematodes may follow a simple evolutionary path, in which division of labour can evolve in a gradual manner as illustrated in Fig. 2. The starting point is a trematode colony in which all individuals are roughly equal: all rediae have similar sizes, produce similar numbers of cercariae per unit time, and spend roughly the same amount of time and energy attacking a competing sporocyst colony from another trematode species. The individual rediae are only roughly equal, however, as perfect similarity is highly unlikely due to epigenetic or micro-environmental effects. Small differences in behaviour and body size among individuals can then be gradually amplified by selection if this benefits the colony. For a proportion of individuals, smaller size improves mobility, and thus access to competitors. Other individuals use more host resources to reach larger sizes and increase their cercarial output. Colony size (total number of rediae) and biomass remain the same throughout, it is only the variation in body size and roles performed among individuals that change. If each increasing degree of division of labour (from a to b, and from b to c in Fig. 2) is associated with an increase in colony fitness (*R*_0_), full division of labour can evolve gradually.

The above shows a possible evolutionary path toward distinct castes; a slightly different strategy can explain the age-structured division of labour observed in some trematode species [12]. In social insects, the formation of functionally specialised castes is often tightly coupled with developmental processes, an age-dependent caste allocation mechanism known as polyethism [24]. This sort of ontogenetic division of labour may exist today in some trematodes, but could also have gradually evolved into distinct, life-long castes. Thus, if in the ancestral state, young rediae performed a mostly defensive role before growing into reproductive rediae, perhaps selection favoured the loss of the reproductive phase in a proportion of individuals, assigning them a permanent non-reproductive role.

On a proximate level, several fundamental questions remain unanswered regarding the within-snail, generation-to-generation development of castes and colony structure from mother rediae to daughter rediae. These are intricately linked to the evolution of division of labour and will need to be investigated using experimental infection and *in vitro* culture. In contrast, the scenarios outlined above could be tested using a comparative approach once data have accumulated on further trematode species presenting different degrees of division of labour.

## Functional adjustments in caste ratios

Despite much research on eusocial insects, crucial aspects of the evolution of division of labour remain poorly understood. These include the identity and importance of factors shaping caste ratios of entire colonies, and of factors modulating behavioural plasticity of individuals within castes [25–29]. These are crucial determinants of the efficiency and resilience of colonies in the face of both immediate threats and long-term changes in environmental conditions. Our research has used the trematode *Philophthalmus* sp. as a model species with division of labour to explore these key aspects of the dynamical nature of social organisation.

Although an intact adult that would allow species description and naming is yet to be obtained, the life-cycle of *Philophthalmus* sp. (hereafter just *Philophthalmus*) has been resolved using genetic markers and experimental studies [30, 31]. Adult worms live in the eyes of seagulls, *Larus* spp., where they mate and release eggs that fall off the birds in tear drops onto intertidal sediments. There, they hatch and the larval stages (miracidia) seek and infect the snail first intermediate host, *Zeacumantus subcarinatus*. Within the snail, from each miracidium asexual multiplication gives rise to a clonal colony of rediae. Cercariae produced by the colony exit the snail and swim briefly in search of a suitable substrate on which to encyst and await ingestion by a suitable bird definitive host; the external surfaces of various gastropods seem to be their preferred encystment substrate [32]. Within the snail, the colony displays division of labour, consisting of large reproductive rediae, and soldier rediae that are one order of magnitude smaller [7] (Fig. 1). Several other trematode species also use the snail *Z. subcarinatus* as their first intermediate host, and thus potentially compete with *Philophthalmus* when co-occurring within the same individual snail. Among these, *Maritrema novaezealandense* (Microphallidae; hereafter just *Maritrema*) is by far the most prevalent, infecting almost half of the snails in some *Z. subcarinatus* populations and frequently co-infecting the same individual snails as *Philophthalmus*. The long-term cercarial output of *Philophthalmus* colonies is reduced by about 50% when they share their snail with *Maritrema* [33, 34], therefore competition has huge fitness impacts for *Philophthalmus*.

In theory, selection at the colony level should favour ratios between different castes that are not fixed but instead capable of adjusting to changing conditions over the lifespan of the colony [25, 35, 36]. For instance, relative investments into different castes should respond to fluctuations in environmental factors like competition and resource availability [25–27, 36]. This prediction has been confirmed experimentally in social insects [37, 38]. In social trematodes, maintaining a small number of soldiers may provide a preventive strike force against possible invasion by competitors. However, excess production of soldiers when the colony is not under threat may come at the expense of cercarial production; with total colony size constrained by host resources, this trade-off suggests that there must be a threat-dependent optimal caste ratio. Thus, repeated exposure to invaders (i.e. other parasites entering the snail host) or actual competition from an established competitor colony within the snail should induce a shift in caste ratios towards more soldiers. Similarly, other threats to the colony may also result in caste ratio adjustments. The snail host can be invaded by microbial pathogens that may jeopardise its survival, and thus that of the colony. If soldiers can also eliminate these microbial invaders, then microbial infection of the snail host may also induce a shift in caste ratios towards more soldiers. Finally, external abiotic conditions may also change and affect the host and in turn the colony’s long-term survival. Under severe conditions, we might expect a shift of caste ratios in the opposite direction, i.e. towards more reproductives, if the host’s longevity is compromised and soldiers cannot improve it.

**Fig. 3:**
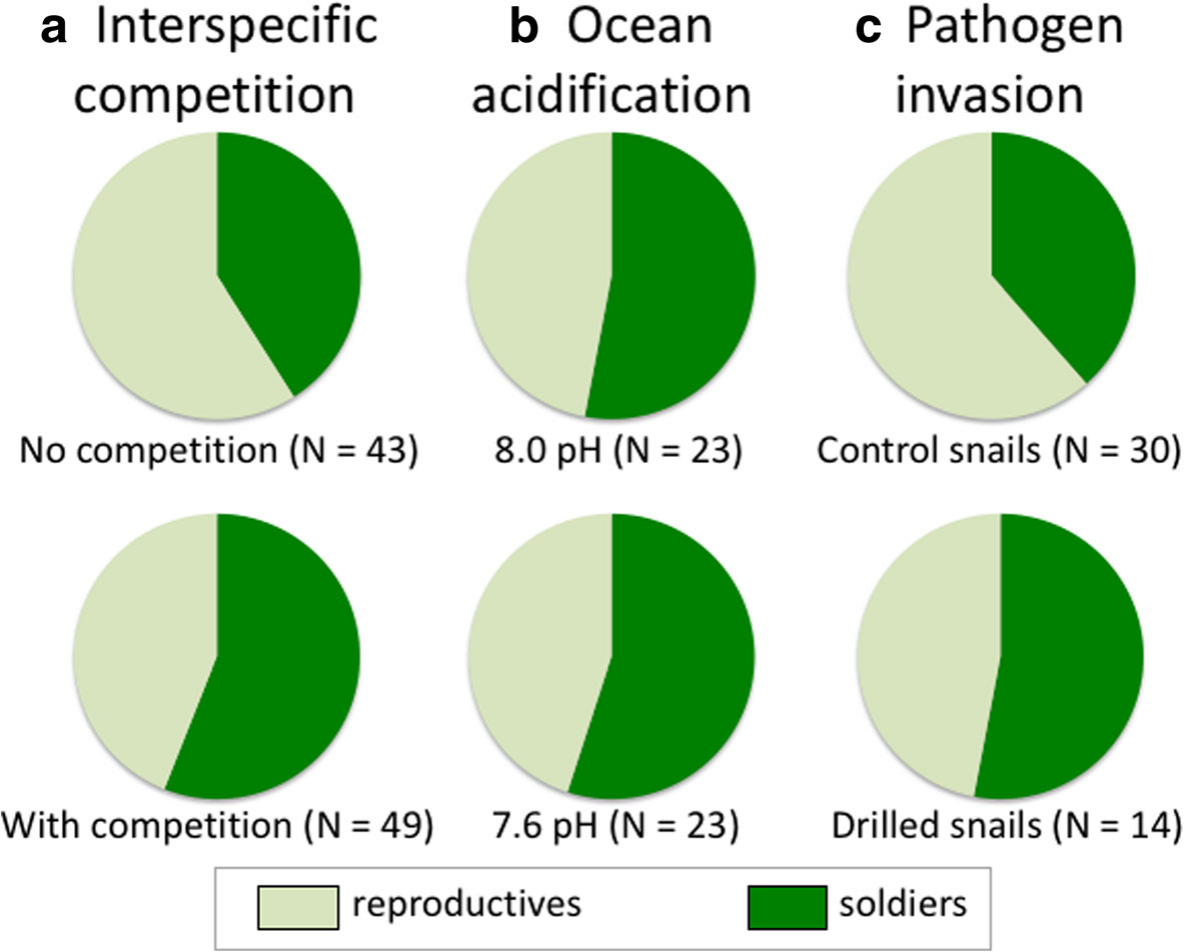
Relative numbers of reproductive and soldier rediae in colonies of the trematode *Philophthalmus* sp. inside their snail host *Zeacumantus subcarinatus*. Each panel contrasts control colonies with those exposed to a different stressor: interspecific competition from *Maritrema novaezealandense* (**a**), ocean acidification (**b**) and a drilled snail shell allowing pathogen invasion (**c**). Numbers of colonies in each group are shown in parentheses. Data from [33, 43, 44]

We tested several of the above predictions using long-term *in vivo* experiments in which *Philophthalmus* colonies are maintained within their snail host under a range of conditions or stressors. After a year under controlled laboratory conditions, the caste ratio of *Philophthalmus* colonies sharing a snail with the competitor *Maritrema* was much more soldier-biased than that of *Philophthalmus* colonies not under competition (Fig. 3a). Thus, *Philophthalmus* colonies raise a greater soldier army when facing a threat. These results come from naturally infected snails that were brought to the laboratory with their existing infections. A similar shift in caste ratio was observed in *Philophthalmus* colonies whose snail host was invaded by *Maritrema* following experimental exposure to *Maritrema* eggs, compared to *Philophthalmus* colonies whose snail host was not invaded [34]. These results support earlier findings [39] and are also aligned with findings on different trematode species with division of labour [40]. Caste ratio adjustments appear to be effective at eliminating the competitor: the size of *Maritrema* colonies (number of sporocysts) is significantly negatively correlated with the number of soldiers in the *Philophthalmus* colony sharing their snail [34].

Interestingly, comparisons among different snail populations from distant localities support the role of competition in shaping *Philophthalmus* caste ratios. In snail populations where the prevalence of *Maritrema* and other potential competitors is very low, *Philophthalmus* colonies display caste ratios that are generally much more biased toward reproductive rediae than those from localities where snails experience prevalent parasitism from other trematode species [41]. This pattern suggests that investment in defense, i.e. the production and maintenance of many soldiers, is locally adapted to match the actual probability of competition. However, the use of avian definitive hosts maintains high genetic connectivity of *Philophthalmus* populations among distant localities [42]. Therefore, the match between caste ratios and local competition threat may result more from plastic adjustments in response to the frequency of invasion attempts by competitors, than from local adaptation in the strict sense.

In the above studies, *Philophthalmus* colonies are pitted against a competitor species, *Maritrema*, whose own colonies consist of sporocysts that lack a mouth. How they would fare against interspecific competitors with rediae, which are generally stronger competitors [23], remains to be determined. And what about intraspecific competition? Can *Philophthalmus* colonies adjust their caste ratios in response to competition from members of their own species? Using microsatellite markers, we showed that a small proportion of snails harbouring *Philophthalmus* have in fact two or more distinct colonies, i.e. distinct clonal lineages issued from different miracidia [43]. Intraspecific competition has a measurable impact on these colonies, as the sizes (total number of rediae) they achieve are lower than the average colony size in absence of competition. However, caste ratios do no differ between *Philophthalmus* colonies experiencing intraspecific competition and *Philophthalmus* colonies on their own [43]. There are at least three possible explanations for this finding: *Philophthalmus* soldiers may be incapable of distinguishing kin from non-kin, soldiers may be ineffective against members of their own species, or intraspecific warfare may lead to lower fitness for both colonies than non-aggression, and therefore natural selection has not favoured caste ratio adjustments under intraspecific competition.

Finally, we explored how *Philophthalmus* colonies adjust their caste ratio when faced with stressors other than competition. We first investigated the effect of starvation of the snail, to determine whether a shortage of resources would shift caste ratios. After a 10-week starvation period, caste ratios of *Philophthalmus* colonies in starved snails were not significantly different from those of colonies in well-fed snails [39]. Then, we exposed *Philophthalmus*-infected snails to simulated ocean acidification, conditions that lead to greater shell dissolution and physiological stress for the host [44]. In slightly acidified conditions, i.e. 7.8 pH, *Philophthalmus* colonies showed a slight tendency toward more soldier-biased caste ratios than under normal conditions (8.0 pH); however, under truly acidified conditions (7.6 pH), caste ratios of *Philophthalmus* colonies were no different from those of colonies at normal pH (Fig. 3b). One explanation for these results may be that when host survival is compromised by either starvation or ocean acidification, *Philophthalmus* colonies maintain their basal caste ratio. Shifting toward more reproductive rediae may be impossible due to space and resource constraints preventing further increases of these large individuals.

In addition to competitors, soldiers may also be capable of eliminating microbial pathogens (bacteria or fungi) that invade their snail host. To test whether *Philophthalmus* colonies adjust their caste ratio when faced with a greater threat of microbe invasion, we drilled holes in the shells of infected snails to provide an entry point for pathogens, and maintained both drilled snails and undrilled control snails for 12 weeks in natural seawater, after which the caste ratios of the *Philophthalmus* colonies they harboured were quantified [45]. We observed a shift toward significantly more soldier-biased caste ratios in *Philophthalmus* colonies within drilled snails than those in control snails (Fig. 3c). The magnitude of the caste ratio adjustment was comparable to that observed in response to interspecific competition (Fig. 3a), suggesting that it may also be a response to a perceived threat against which soldiers are effective. Of course, the ability of soldiers to control or eliminate microbial pathogens remains to be demonstrated, and the above finding may instead be a response to the stress incurred by the host from drilling.

Overall, these *in vivo* studies have demonstrated that *Philophthalmus* colonies are responsive to potential threats, and that colony structure can change accordingly. The parallels with caste ratio adjustments in social insect colonies are striking [37, 38], and it will be interesting to see whether they occur more widely across all trematode species with division of labour among distinct castes.

## Phenotypic plasticity of caste members

The second key aspect we explored regarding the social structure of trematode colonies was the phenotypic plasticity of individuals within each caste. Caste ratio adjustments take time, because they require the production and turnover of individuals, a process that can take weeks or months. Rapid responses to changing conditions would instead require individual plasticity of caste members: individuals specialised for one function adopting other functions when required. For instance, under intense competition, individuals specialised for reproduction might adopt defensive roles, especially if there is a shortage of individuals in the soldier caste. The extent and causes of individual variability and plasticity within castes remain poorly understood, even among the well-studied social insects [46, 47].

To investigate the plasticity of caste members in the trematode *Philophthalmus*, we first needed to develop an *in vitro* culture system that would enable repeated observations of individual rediae. Our culture medium allows us to extract rediae from their snail host and maintain them alive and producing cercariae for several weeks [48]. Small subsets of rediae from each snail can be chosen to produce mini-colonies with given caste ratios, that are then maintained under different conditions in wells of tissue culture plates, for instance with or without the competitor *Maritrema*. Importantly, because many mini-colonies with the same caste ratio can be extracted from the same snail and allocated in equal numbers to the different treatments, we can control for any genetic influences and isolate the pure effect of the treatment conditions on the plastic growth or behaviour of individual rediae.

First, we investigated the behavioural plasticity of reproductive rediae as a function of the number of soldiers per mini-colony. Reproductives are capable of defensive actions, by attaching to competitors and feeding on them. In a short-term *in vitro* experiment, the fewer soldiers were present, the greater the proportion of time spent by reproductives in contact with the competitor *Maritrema* [49]. The allocation of time toward defense made by reproductive rediae therefore depends on the social context: when there is a shortage of soldiers, the reproductives adjust their behaviour to assume a greater defensive role. This plastic response can serve to mitigate the fitness impact of competition during the period needed for the colony to readjust its caste ratio.

Secondly, we asked whether soldiers can adopt a reproductive role if cultured in mini-colonies in which reproductives are either rare or absent. The short answer is no, they appear incapable of doing so. Soldier rediae show some variation in body sizes in *Philophthalmus* colonies within snail hosts, and they can grow larger by 37% after 28 days when reared *in vitro* with competitors present than in the absence of competitors, possibly as a consequence of the extra resources they obtain by feeding on the competitor [50]. However, when cultured in absence of reproductives, even after 50 days soldiers only showed modest growth, still remaining about an order of magnitude smaller than reproductives, and few showed signs of developing the germinal mass necessary for the production of cercariae (C. Lagrue and R. Poulin, unpublished data). This suggests asymmetrical plasticity among members of the two castes: *Philophthalmus* colonies appear capable of compensating for a loss of soldiers through the phenotypic plasticity of reproductives, but not for a short-term lack of reproductives.

The above *in vitro* approach also served to demonstrate the existence of a trade-off between reproduction and defense in *Philophthalmus*, as well as quantify the cost of maintaining an army of soldiers that do not directly contribute to the colony’s fitness. The cercarial output of individual reproductive rediae was shown to decrease significantly as a function of the proportion of their time spent in defensive interactions with competitors [49]. This trade-off supports one of the key assumptions of the evolutionary model presented earlier for the origin of division of labour in trematodes. In addition, our studies of *Philophthalmus* mini-colonies cultured *in vitro* indicate that in the presence of the competitor, higher caste ratios, i.e. a greater number of soldiers for a fixed number of reproductives, result in both larger-bodied reproductive rediae [50] and greater total cercarial output for the mini-colony [51]. However, in absence of competition, an excess of soldiers has no clear benefit for the colony, but also no obvious fitness costs [51]. The much smaller body size of soldiers means that they require relatively few host resources to maintain in absence of competition. They are therefore not costly to maintain when not needed, as a cheap insurance against potential invasion by competitors or pathogens, but their presence really pays off when they are needed.

## Future directions

In addition to consolidating research on the basic themes raised in this review, several other key issues remain to be explored in social trematodes. For instance, we still do not know if and how the two castes communicate with each other. Members of one caste most likely rely on tactile, chemical or other forms of stimuli to sense the presence and/or abundance of the other caste and respond accordingly. Also, we are yet to identify the proximate cues used by soldiers for the recognition of kin, non-kin and enemies such as interspecific competitors.

In addition, from a developmental perspective, phenotypic divergence in morphology and behaviour between functional castes in social organisms is typically a result of differential gene expression [24, 52–54]. Indeed, the fact that genetically identical individuals develop into phenotypically distinct castes is increasingly explained via epigenetic mechanisms [55]. DNA methylation, which affects the availability of genes for transcription, is a key epigenetic mechanism through which caste determination is achieved in various groups of social insects [55]. The role of methylation in caste differentiation may be tested experimentally using a histone deacetylase inhibitor previously used to alter the development of trematode parthenitae in gastropod intermediate hosts [56]. Therefore, at the epigenetic level, specific mechanisms involved in caste differentiation may be explored following recent developments in the insect literature, to determine whether caste differentiation mechanisms are similar in widely divergent social organisms.

## Conclusions

Division of labour has evolved independently on more than one occasion in trematodes, each time leading to a clear partitioning of defensive and reproductive functions between two morphologically distinct castes. The ratio between these castes can change adaptively within weeks in response to interspecific competition or other environmental pressures. In contrast, the plasticity of individual caste members is much more limited, and consists mostly of reproductive rediae assuming a greater defensive role in the absence of soldiers. These observations show both parallels and differences when compared to division of labour in social insects, for which much more is known. Social trematodes offer several conceptual and logistical advantages over traditional social insect model systems for the study of division of labour and caste specialisation. First, trematodes form asexual clonal colonies, thus avoiding any potential conflict of genetic interest that can arise in haplodiploid social insects with sexual reproduction. Secondly, trematodes form only two distinct morphological castes, which represents one of the simplest social systems known, thereby facilitating tests of fundamental theories of social evolution. Thirdly, trematode colonies live in gastropod hosts that are abundant, easy to collect, long-lived and easy to maintain under laboratory conditions; it is therefore straightforward to obtain *in vivo* and ecologically realistic estimates of reproductive output and colony demography across time and under various environmental conditions. Finally, it is also possible to maintain rediae *in vitro*, allowing experimental manipulation and observation at the level of colony subsets or individuals. Trematodes with division of labour provide a great platform for an exciting collaborative research program among a broad range of disciplines, extending beyond parasitology to genetics, developmental biology and evolutionary ecology.
